# Barker Hypothesis and Hypertension

**DOI:** 10.3389/fpubh.2021.767545

**Published:** 2022-01-21

**Authors:** Felix Jebasingh, Nihal Thomas

**Affiliations:** Department of Endocrinology, Diabetes and Metabolism, Christian Medical College, Vellore, India

**Keywords:** low birth weight, Barker hypothesis, Brenner hypothesis, low nephron number, hypertension

## Abstract

Early onset hypertension is one of many major medical disorders that have evolved over the current millennium across both the developing as well as the developed world. Though various mechanisms have been postulated for the evolution of hypertension in these individuals, one of the most relevant ones is that of low birth weight and its association with hypertension. Barker from historical evidence has postulated the foetal onset adult disease (FOAD) or Thrifty phenotype on Low Birth Weight (LBW) associated hypertension. Later, Brenner highlighted the importance of low nephron mass and future implications. In this review we elaborate the mechanisms that were postulated for LBW-related hypertension as well the potential antihypertensive therapy that may be used in these individuals.

## Introduction

Hypertension is a leading cause for the global burden of disease across developed and developing countries. Moreover, it is an independent risk factor for the subsequent evolution of coronary artery disease. The prevalence of hypertension among the Indian population has been rising since the turn of the century. This may be attributed to the increase in sedentary lifestyle pattern and increase in body weight (1).

The transition as a result of the free market economic reforms that occurred toward the latter part of the twentieth century, has increased the availability of high calorie food across urban and rural India (2). As per the Fourth National Family Health Survey (NFHS-4), the prevalence of hypertension is as high as 20 percent in most of the rural India (3, 4).

Having said that, it is important to think tangentially of other potential precipitating factors. According to NFHS-4 survey, 18% of new born infants fall into the category of Low Birth weight (LBW) as the per the World Health Organisation (WHO) criteria i.e., birthweight <2.5 kg.

## Low Birth Weight Cohorts Across the Globe

The *Helsinki and Hertfordshire cohorts* that were followed up between the 1930s and 1940s, with over 20,000 sample population has shown clear evidence linking poorly developed foetal growth with metabolic syndrome in their adult life (5). Moreover, these effects may be transmitted to subsequent generations as having an increased prevalence of diabetes mellitus, hypertension, and coronary artery disease (6).

However, those foetuses subjected to the *Leningrad famine* during World War II, has shown a decreased prevalence of diabetes, hypertension as well other metabolic problems, after they were on a continuous calorie restriction during their infancy. This is in contrast to the Dutch counterpart that was followed during the early part of twentieth century. These contrasting scenarios in association with low birth weight and the metabolic syndrome have provided a different dimension toward the pathogenesis of the metabolic syndrome and birth weight (7).

Subsequently in 1962, James V Neel proposed the thrifty-genotype hypothesis, stating that “an individual's adaptation to the environment was dependent on genes selected over a long period of time.” It proposed that genes that favour survival during prolonged adverse environment in the foetal life, can be detrimental if those same genes are subjected to a state of surplus energy at a later part of life, as was shown in Hertfordshire cohort (8). This was subsequently labelled as the thrifty genotype hypothesis.

**Figure 1:**
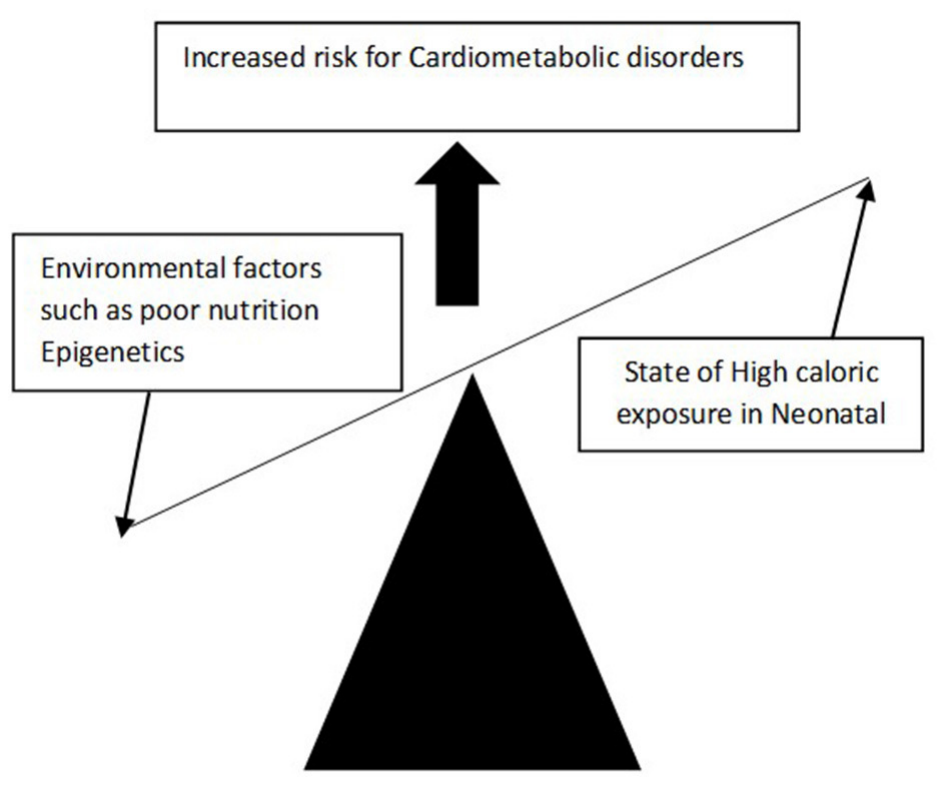
Barker's hypothesis.

However, Hales and Barker later challenged this theory and proposed that the foetus which experiences suboptimal nutritional uptake during intrauterine development, may lead to reprogramming of foetal genes that subsequently alters foetal structure, function as well as metabolic changes. This hypothesis is called the FOAD (Foetal origin of adult disease) or thrifty phenotype or Barker's hypothesis or “developmental origins of adult health and disease” hypothesis (DOHAD) (Figure 1). The FOAD hypothesis represents a mismatch between foetal life and neonatal life, thereby increasing the risk for cardiometabolic diseases. Hence low birth weight, which is a surrogate marker of poor foetal growth, is linked to hypertension, diabetes, obesity, and insulin resistance. In addition, a disproportionate catch-up fat growth, in comparison with lean body mass, is one of the major driving factors for the development of cardiometabolic problems among adults with LBW.

In the Indian context of low birth weight and cardiometabolic consequences, studies from Vellore, Pune, and Delhi has highlighted an increased prevalence of cardio-metabolic disorders amongst those born with LBW (9–12).

One of the major risk factors for the low birth weight is maternal age. A U-shaped relationship has been described between maternal age and LBW. Teen age pregnancies on one hand and an increased maternal age on the other hand have been shown to be associated more with LBW (13). Moreover, untreated sexually transmitted disorders such as chlamydia and bacterial vaginosis have been known to precipitate LBW. Moreover, the literature has also cited domestic violence during pregnancy to be associated with LBW (14). Substance abuse such as alcohol, smoking, or using illicit drugs such as heroin or cocaine are also found to be associated with LBW (15). Maternal educational status below the primary school cut off, a reduced birth interval of <2 years, poor maternal weight gain (<4 kg), pregnancy induced hypertension and poor antenatal follow ups have been shown to be associated with LBW in a study done in Southern India (14–16).

Hence, LBW might be prevented through regular antenatal checkups and appropriate therapy for sexually transmitted diseases during pregnancy. More so factors such as teen age pregnancy and substance abuse during pregnancy lead to LBW.

## Hypertension Amongst Low-Birth-Weight Individuals

David Baker has demonstrated that low birth weight could cause raised blood pressure in adult life and this has already been established in many studies from different ethnicities. Therefore, lower the birth weight, higher the risk of adult onset of hypertension (13–16). A metanalysis on LBW and hypertension comprising nine studies has shown that there was an odds ratio of 1.21 (95% CI 1.13, 1.30) in developing hypertension among those born with LBW (17).

A retrospective study from Sri Lanka (*N* = 122), that surveyed the hospital records of low birth weight in relation to hypertension in adulthood, found a significant association with high systolic Blood pressure (OR = 2.89; 95% CI: 1.01, 8.25), and hypertension (OR = 3.15; 95% CI: 1.17, 9.35; *P* = 0.03) and no association with diastolic blood pressure after adjusting for other independent factors that may determine adult-onset hypertension (18). Law et al. in his metanalysis has also demonstrated an inverse relationship with birth weight and hypertension in adults (19).

## Low Birth Weight, Catch Up Growth, and Blood Pressure

Studies have provided enough evidence about the association between LBW in relation to the timing of a foetal nutritional insult during the stages of development. A thin infant at birth with a low Ponderal index is more likely to have had a sustained duration of a nutritional insult in the last trimester. However, a neonate with a small head circumference may have had a nutritional insult through all three trimesters (20). A study by Thomas N et al. has shown the presence of a borderline trend toward high diastolic blood pressure amongst an Asian Indian Low birth weight cohort (12).

It is important to understand that there are two types of catch-up growth in childhood, i.e., skeletal and non-skeletal growth. Skeletal catch-up growth implies the acceleration in growth following a health crisis or illness, to eventually achieve a reasonable final height. Non-skeletal growth implies: either the weight gained or body mass index (BMI) accrued in relation to the baseline birth weight.

A systematic review of 80 studies has shown that there is an inverse association between birth weight and SBP in adults among those with a high catch-up growth during childhood. However, the review failed to show a significant association between the Ponderal index and high blood pressure. This may suggest that an individual with a sustained intrauterine nutritional insult and a significant non-skeletal catch-up growth during childhood is also at risk for future hypertension (16, 21).

## Pathogenesis of LBW and Hypertension

There are several mechanisms that have been proposed for the association between Low Birth weight and hypertension.

One proposed mechanism was that an increased pressure in foetal circulation, as a compensatory mechanism in maintaining placental perfusion, might persist even after birth (22). Another mechanism that has been proposed: intrauterine growth retardation causing low birth weight may lead to accelerated postnatal growth and thereby, an accelerated rise in blood pressure. This was shown in both mothers with and without hypertension during pregnancy (15, 23).

Gunhild Keller performed autopsy studies and demonstrated that reduced nephron numbers with associated hypertrophy of the glomeruli was common among those with systemic hypertension compared with those without hypertension (median, 6.50 × 10^3^/mm^3^ vs. 2.79 × 10^3^/mm^3^) (24).

Evidence from native Australian Aborigines have demonstrated that there is a strong association between reduction in the nephron numbers and an increased prevalence of adult related hypertension and cardio-renal disorders (25). In addition, this study has also shown that there is a strong link between low birth weight, low nephron numbers, and hypertension at adulthood.

The results from the Dutch famine birth cohort has shown that the variations in presentation were dependent on the type of metabolic disorder and the timing of the intrauterine insult. Those with a first trimester insult resulted in an increased risk of coronary artery events (OR 3.0, 95% CI 1.1–8.1), whereas an insult during the second trimester had shown an increased prevalence of microalbuminuria and a further increase in systolic hypertension (OR 2.1; 95% CI, 1.0–4.3) (26). Amongst those with mid or a third trimester insult, the impact involved an increased prevalence of dysglycemia during adulthood (27).

There was no association between the type of specific nutritional deficiency in foetal life and the onset of hypertension in adulthood. But if the protein intake is restricted to <5 percent it can be one of the major factors for its association (28).

The INTERSALT study has shown that a subject taking a larger quantity of salt and a prolonged ingestion of salt over years, has a greater propensity to develop hypertension, when compared with those counterparts taking diet with low or normal salt intake (29). As per the studies, the average intake of salt is about 11 g per day, which is more than double the WHO's recommended maximum intake of 5 g per day (30). Therefore, a two-hit hypothesis has been proposed by Thomas et al. in their work on the development of hypertension amongst those born LBW (12). The factors that have been proposed in the two hit hypotheses with regards to low birth weight and hypertension include, reduction in the number of nephrons and a subsequent decline in glomerular functions and a high intake of salt when compared to the western population.

According to the Borst Guyton concept, chronic hypertension is due to the imbalance in glomerular pressure and sodium homeostasis in the kidneys (31). The reduced critical mass of nephrons imposes immense workload on the individual nephrons by increasing hyperfiltration. Furthermore, glomerular sclerosis in adult life causes nephron death, thereby initiating a vicious cycle and thereby resulting in end stage renal disease (32). Moreover, obesity increases renal filtration load and the associated insulin resistance further augments the workload on the kidneys. Hence, the imbalance between the triad of low birth with progressive weight gain, reduced nephron mass and an increased load on the kidneys and its related sodium homeostasis induces an early onset hypertension in those born with LBW (33).

**Figure 2:**
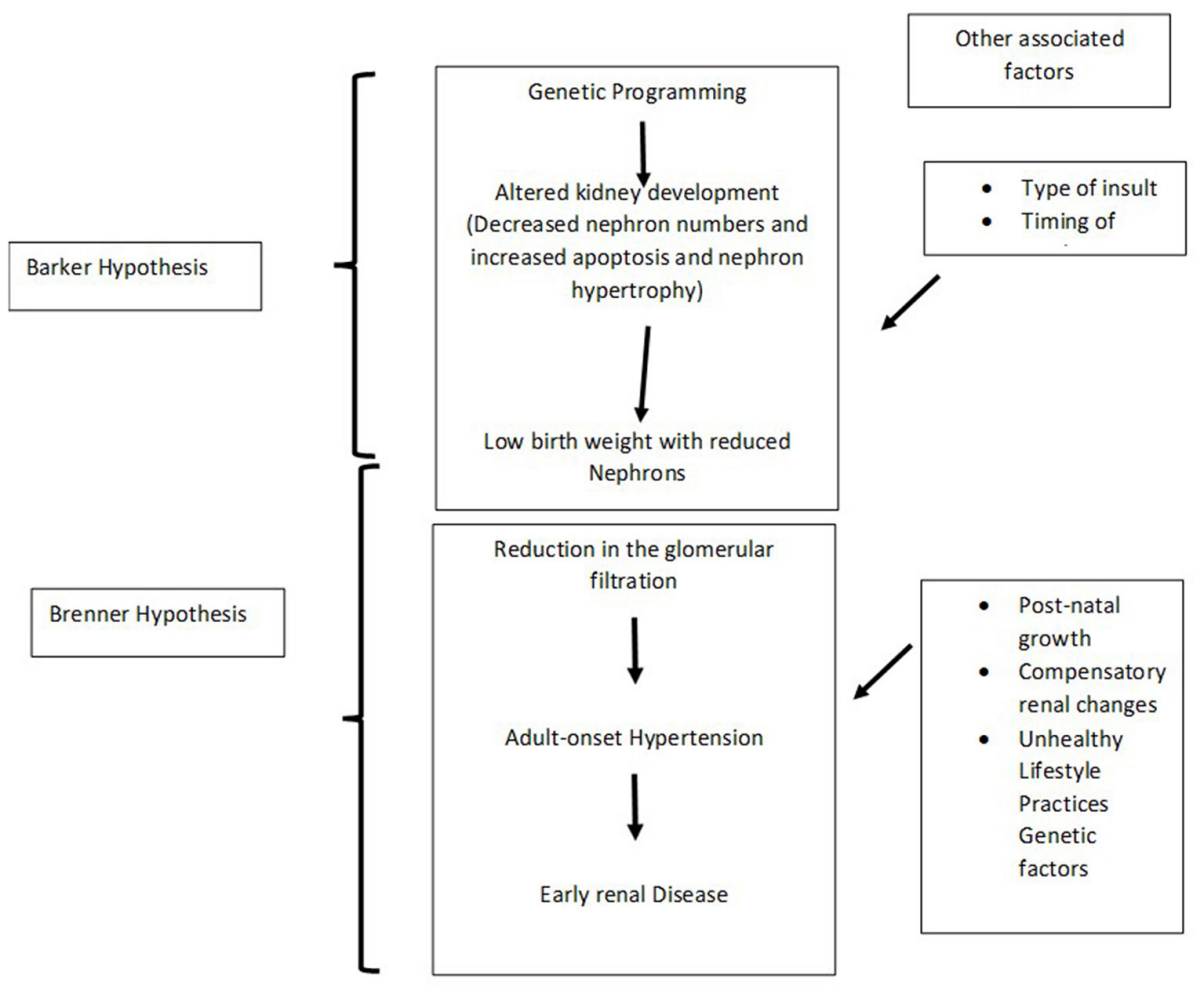
Integration of the Barker and Brenner hypothesis.

The Brenner hypothesis in conjunction with the Barker hypothesis may help interpret the association between the pathogenesis of hypertension amongst individuals born with LBW (27, 34) (Figure 2).

## Progression of Hypertension With Age

There is a clear association between arterial compliance, elastogenesis and hypertension (35). It was proposed by Barker that there is decreased elastin production among neonates with LBW due to poor vasculogenesis *in vitro*. This alteration decreases the arterial compliance that causes hypertension in individuals with LBW (15).

## Clinical Implications

One should diligently ask for a history of low birth weight among those with obesity, early onset hypertension, diabetes, or coronary artery disease as LBW is a major contributor for the development of cardiovascular diseases (36). The most appropriate way to obtain this is often not from the subject concerned, but the mother of the subject who may be available to give the history of low birth weight.

Singhal et al. followed-up infants with LBW who were given a specific diet for 1 month, after 20 years and found that those who were given a high carbohydrate and high fat diet had elevated pro-insulin levels, suggesting these young adults might develop diabetes in future. Thus, even a brief duration of a nutritional intervention in early infancy may have a major stake in changing the prevalence of future diabetes (37). Lowering the systolic and diastolic blood pressure by 10- and 5-mm Hg, respectively, reduces cardiovascular risk at 65 years of age by 25 percent and strokes by 35% (38).

After analysing 354 trials, Law and Ward demonstrated that a combination of three antihypertensives at half of their standard dose, reduce two-third of strokes, and half the number of CAD at 60 years of age. Moreover, at the lowest possible doses, these antihypertensives has negligible adverse effects (39). There is evidence of increased glucocorticoid sensitivity in patients with LBW. Moreover, glucocorticoid intake during pregnancy may also induce foetal growth retardation. Evidence suggests that those children who were exposed to glucocorticoids have a subsequently higher prevalence of hypertension. The proposed mechanism is that the glucocorticoids increase the sensitivity of angiotensin converting enzymes and thereby increasing the levels of angiotensin -II. This elevated intraglomerular angiotensin may induce hypertension (40).

The role of the renin-angiotensin-aldosterone pathway as well as early onset proteinuria among those with LBW suggests that ACE-inhibitors or Angiotensin receptor blockers could be a potential medication involved in the therapy of individuals with LBW related adult-onset hypertension (41–45).

Though there are many studies on LBW and metabolic problems across the globe, the studies having LBW as the theme are very limited, in developing countries like India and other African countries. With an increase in the prevalence of Hypertension amongst the developing countries, the cause for early onset hypertension could be multifactorial rather than LBW as a sole factor. More so, the antihypertensives that could be used as first line medications in patients with LBW and early onset hypertension have not been clearly elucidated as yet (46, 47).

## Conclusion

Many studies have clearly mentioned the association between low birth weight and the subsequent risk of hypertension. This has been demonstrated both in obese as well as non-obese adults. There are several mechanisms that have been postulated in early hypertension among those who were born with LBW. A concise Barker and Brenner hypothesis explains the cause of hypertension in individuals born with LBW. According to the Barker hypothesis, reprogramming of genes in the foetus due to suboptimal nutrition in intrauterine life induces a functional and structural change in the foetus, subsequently leading on to various illnesses, such as hypertension, diabetes, and obesity; particularly when there is unrestricted or increased calorie intake during the neonatal period. Brenner hypothesised that LBW babies tend to have reduced critical nephron related mass that induces a mismatch in the sodium homeostasis in the glomeruli between foetal and adult life due to work load on the kidneys. This leads to an early onset hypertension among those born with LBW. Hence birth weight is inversely proportional to adult-onset hypertension. However, there is no definitive evidence-based research to suggest as to what antihypertensive therapy may be of use for patients with hypertension and who have been born LBW. Physicians should be aware of LBW as a potential cause for early onset hypertension and should elicit this important history from the mother of the patient. Those born with LBW should be provided with adequate nutrition that may suffice for normal linear growth. They are not to be overfed with additional calories, as through the mechanisms described above, it could result in young onset hypertension.

## Author Contributions

FJ and NT wrote and reviewed the manuscript. Both authors contributed to the article and approved the submitted version.

## Conflict of Interest

The authors declare that the research was conducted in the absence of any commercial or financial relationships that could be construed as a potential conflict of interest.

## Publisher's Note

All claims expressed in this article are solely those of the authors and do not necessarily represent those of their affiliated organizations, or those of the publisher, the editors and the reviewers. Any product that may be evaluated in this article, or claim that may be made by its manufacturer, is not guaranteed or endorsed by the publisher.
